# From Polyphenols to β-Lactamases: Multitarget Strategies to Defeat Severe Resistance

**DOI:** 10.3390/ijms27062702

**Published:** 2026-03-16

**Authors:** Michele Nappa, Emanuela Santoro, Roberta Manente, Angelo Cianciulli, Giuseppina Moccia, Francesco De Caro, Mario Capunzo, Giovanni Boccia

**Affiliations:** 1 Department of Medicine, Surgery and Dentistry “Scuola Medica Salernitana”, University of Salerno, 84081 Salerno, Italy; mnappa@unisa.it (M.N.); ancianciulli@unisa.it (A.C.); gmoccia@unisa.it (G.M.); fdecaro@unisa.it (F.D.C.); mcapunzo@unisa.it (M.C.);; 2 San Giovanni di Dio e Ruggi d’Aragona University Hospital, 84081 Salerno, Italy; 3 Public Health Laboratory for the Analysis of Community Health Needs, Department of Medicine and Surgery, University of Salerno, Baronissi Campus, 84081 Baronissi, Italy; 4 Integrated Care Department of Health Hygiene and Evaluative Medicine, San Giovanni di Dio e Ruggi d’Aragona University Hospital, 84131 Salerno, Italy; 5 Hospital and Epidemiological Hygiene Unit, San Giovanni di Dio e Ruggi d’Aragona University Hospital, 84131 Salerno, Italy

**Keywords:** antimicrobial resistance, antibiotic adjuvants, resistance determinants

## Abstract

Antimicrobial resistance (AMR) is one of the most pressing global public health challenges, compromising the effectiveness of standard antibiotic therapies and increasing morbidity, mortality, and healthcare costs. The scarcity of new antibiotics has driven research into alternative strategies to restore or enhance the effectiveness of existing drugs. Natural compounds, including polyphenols, alkaloids, terpenes and terpenoids, antimicrobial peptides, and microbial secondary metabolites, exhibit multitarget activities such as membrane disruption, efflux pump inhibition, biofilm suppression, and quorum sensing interference. In parallel, synthetic and semi-synthetic small-molecule inhibitors have been rationally designed to target specific resistance determinants, including β-lactamases, efflux systems, quorum sensing pathways, and stress-induced mutagenesis mechanisms such as the SOS response and DNA repair processes. These agents act as adjuvants, restoring susceptibility or reducing bacterial virulence without exerting strong selective pressure. The integration of natural bioactive compounds and targeted small-molecule inhibitors represents a promising complementary strategy for conventional antibiotics. Further pharmacological and clinical investigations are required to translate these approaches into effective tools within antimicrobial stewardship programs and broader public health strategies aimed at mitigating the global burden of AMR. This narrative review analyses the recent literature on natural compounds and synthetic or semi-synthetic small-molecule inhibitors with documented activity against antimicrobial resistance mechanisms.

## 1. Introduction

### 1.1. Antimicrobial Resistance

Antimicrobial resistance (AMR) is a multifactorial phenomenon of major biological and clinical relevance, resulting from the interaction between the high genetic adaptability of microorganisms and the selective pressure exerted by exposure to antimicrobial agents [1]. In this context, bacteria have developed a broad repertoire of molecular strategies that enable survival in the presence of otherwise lethal antibiotic concentrations, substantially compromising the effectiveness of available therapies [2].

A central mechanism involves alteration of the antibiotic’s molecular target. Point mutations, genetic rearrangements, and the acquisition of exogenous genes may lead to structural modifications of macromolecules involved in essential cellular processes such as cell wall synthesis, DNA replication, or protein translation. These variations reduce the binding affinity of the antibiotic for its target without significantly impairing the biological functionality of the bacterial cell, thereby allowing microbial persistence under adverse selective conditions [1,2].

Another widely used strategy is the enzymatic neutralization of the antibiotic. Numerous bacterial species are capable of expressing enzymes that inactivate the drug through hydrolytic reactions or covalent modifications. Among these, β-lactamases represent the most extensively studied paradigm; however, analogous mechanisms have also been described for antibiotic classes other than β-lactams, highlighting the remarkable metabolic versatility of bacteria in counteracting antimicrobial action [1,3].

Resistance may also be mediated by alterations in the intracellular pharmacokinetics of the antibiotic [2].

Changes in outer membrane permeability, often resulting from variations in the expression or structure of porins, limit drug entry into the cell. In parallel, activation or overexpression of efflux systems contributes to the active extrusion of the antibiotic, reducing its intracellular concentration below the threshold required to exert bacteriostatic or bactericidal effects [3].

**Figure 1:**
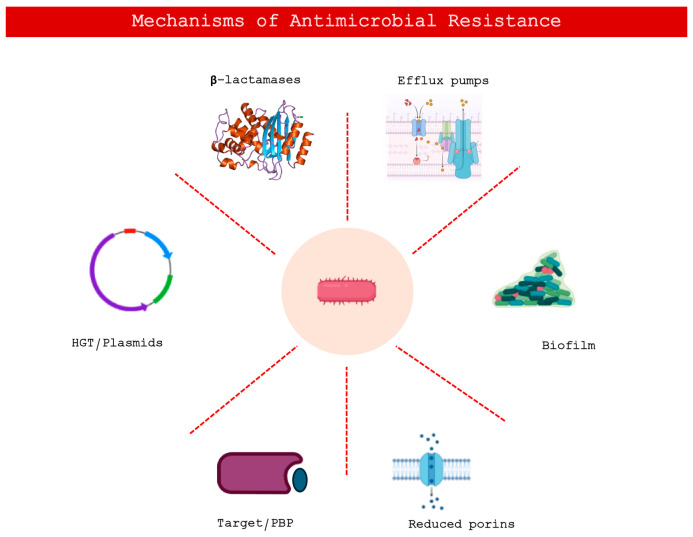
Schematic representation of the primary mechanisms of antimicrobial resistance (AMR) in bacteria. The illustration highlights key strategies employed to evade drug action, including enzymatic degradation (e.g., beta-lactamases), active expulsion via efflux pumps, formation of protective biofilms, reduction in membrane permeability through porin downregulation, modification of molecular targets (e.g., PBPs, Penicillin-Binding Proteins), and horizontal gene transfer (HGT) mediated by plasmids.

A crucial role in the dissemination of resistance is played by horizontal gene transfer. Plasmids, transposons, and integrons act as vectors for resistance genes, promoting their rapid spread within bacterial populations and across phylogenetically distant species. This process accelerates the emergence of multidrug-resistant strains and contributes to the epidemiological complexity of the phenomenon, making the control of resistance particularly challenging in both clinical and environmental settings [4]. Taken together, these mechanisms depict an extremely dynamic and continuously evolving landscape in which antimicrobial resistance emerges as the result of integrated molecular adaptations rather than isolated alterations. A thorough understanding of these processes is therefore essential for the development of new therapeutic strategies and for the implementation of more rational approaches to antimicrobial use [3]. Figure 1 summarizes the main mechanisms of bacterial resistance.

### 1.2. Natural Compounds and Synthetic and Semisynthetic Small-Molecule Inhibitors Against Antimicrobial Resistance

In recent years, natural compounds have attracted increasing attention as potential antimicrobial agents or resistance modulators [5]. Polyphenols, alkaloids, terpenes and terpenoids, natural antimicrobial peptides, and microbial secondary metabolites exhibit a wide range of mechanisms of action, including inhibition of bacterial growth, destabilization of cellular membranes, interference with quorum sensing, and potentiation of existing antibiotics [5,6]. Their structural and functional diversity represents a valuable resource for the identification of novel bioactive molecules capable of overcoming or bypassing resistance mechanisms [6].

In parallel, the development of small-molecule inhibitors targeting specific determinants of antimicrobial resistance has emerged as another promising strategy [7]. These compounds, often designed through rational drug design approaches, can target key elements such as β-lactamases, efflux pumps, target-modifying systems, and regulatory pathways involved in bacterial virulence and persistence. By selectively inhibiting these mechanisms, small molecules may restore antibiotic susceptibility or reduce the selective pressure that promotes the emergence of resistance [7,8].

This review article aims to provide a critical and up-to-date overview of the role of natural compounds and small-molecule inhibitors as potential strategies against antimicrobial resistance [9].

## 2. Discussion

### 2.1. Polyphenols

Polyphenols constitute a broad class of natural compounds found in fruits, vegetables, tea, wine, and spices, known for their antimicrobial activity and their ability to modulate resistance mechanisms in pathogenic microorganisms. These compounds include flavonoids (e.g., quercetin, catechins), stilbenes (e.g., resveratrol), tannins, and phenolic acids, which act through multiple pathways to counteract antimicrobial resistance, often in synergy with conventional antibiotics [9].

One of the main mechanisms by which polyphenols enhance antibiotic efficacy is the inhibition of efflux pumps, which represent one of the most important bacterial resistance systems capable of actively expelling drugs from the cell. Polyphenols such as quercetin, curcumin, luteolin, and epigallocatechin-3-gallate (EGCG) have demonstrated the ability to interfere with the function or expression of efflux pumps, thereby increasing the intracellular concentration of antibiotics and restoring antibiotic susceptibility in resistant strains [10].

Another key mechanism involves interference with biofilm formation and maturation, as biofilms are structured communities that protect bacteria from antimicrobial stress. Several polyphenols, including proanthocyanidins and ellagic acid, have been shown to reduce bacterial adhesion, destabilize the biofilm matrix, and facilitate antibiotic penetration into biofilms formed by multidrug-resistant species [9].

Polyphenols also modulate quorum sensing, the intercellular communication system that regulates the expression of virulence genes and antibiotic tolerance. Classical studies have demonstrated that various phenolic compounds can inhibit quorum sensing pathways in different pathogens, thereby impairing virulence factor expression and the coordinated resistance response [11].

Finally, polyphenols may alter cell membrane permeability, promoting antibiotic uptake and contributing to increased pathogen susceptibility. The combined action of these mechanisms not only directly reduces microbial growth but also enhances the effectiveness of existing antibiotics, paving the way for adjuvant therapeutic strategies based on natural compounds [9].

### 2.2. Alkaloids

Alkaloids are a large class of nitrogen-containing compounds of plant origin, characterized by heterocyclic structures and remarkable chemical diversity, which makes them attractive antibacterial agents and modulators of antimicrobial resistance. Although not always highly microbicidal on their own, these compounds can modulate or counteract key bacterial resistance mechanisms, thereby enhancing the efficacy of antibiotics when used in combination [12].

A well-documented anti-AMR mechanism of alkaloids is efflux pump inhibition. Alkaloids such as reserpine and piperine are capable of interfering with efflux pump activity in both Gram-positive and Gram-negative bacteria, partially restoring antibiotic susceptibility and reducing the minimum inhibitory concentration (MIC) of drugs in resistant strains [12].

Alkaloids may also influence biofilm formation and persistence. Studies on fumiquinazoline-derived alkaloids have demonstrated both direct antibacterial effects and the ability to inhibit biofilm accumulation and interfere with efflux systems associated with biofilm formation, suggesting a dual role of these compounds in reducing resistance [13].

Another mechanism involves interference with essential cellular processes, such as DNA synthesis or cell division. For example, the alkaloid berberine can not only reduce efflux pump activity but also intercalate into bacterial DNA and inhibit enzymes such as DNA gyrase, thereby impairing bacterial replication and enhancing the effect of antibiotics targeting nucleic acids [12].

Finally, some alkaloids exhibit the ability to modulate quorum sensing, which regulates the expression of virulence and resistance genes. Although less extensively studied in the context of alkaloids compared with other classes of natural compounds, alkaloids such as caffeine have been reported to modulate quorum sensing signals, with potential effects on virulence and resistance development [12].

Overall, alkaloids represent a promising class of resistance modulators, acting on both direct resistance mechanisms (such as efflux pumps and biofilm formation) and fundamental cellular processes, with considerable potential in combination strategies with conventional antibiotics to overcome resistant bacterial strains.

### 2.3. Terpenes and Terpenoids

Terpenes and terpenoids constitute some of the most abundant classes of natural secondary metabolites derived from isoprene, and are widely present in the essential oils of many aromatic plants. These compounds exhibit intrinsic antimicrobial activity and the ability to modulate bacterial resistance mechanisms, thus representing potential anti-AMR strategies both as direct agents and as adjuvants to conventional antibiotics [14].

A well-documented mechanism is efflux pump inhibition. A systematic review has highlighted that numerous terpenes, including carvacrol and thymol, act as efflux pump inhibitors in both Gram-positive and Gram-negative strains, demonstrating synergistic effects with standard antibiotics and a reduction in MIC values in resistant strains due to impairment of bacterial efflux systems [15].

Terpenes and terpenoids also influence biofilm formation. In vitro studies on *Acinetobacter baumannii* have shown that sub-MICs of compounds such as carvacrol and thymol, extracted from oregano essential oil, significantly reduce biofilm biomass and motility associated with bacterial adhesion, thereby hindering infection persistence and biofilm-associated resistance [16].

Another relevant mechanism involves modulation of membrane permeability and integrity. Terpenes such as limonene, carvone, and β-citronellol disrupt the lipid structure of membranes in *Escherichia coli* and *Staphylococcus aureus*, inducing leakage of intracellular material and increasing bacterial susceptibility to antimicrobial agents through destabilization of the cellular barrier [17].

Finally, several studies suggest that terpenes may interfere with quorum sensing and other virulence regulatory systems, although this aspect is less extensively characterized compared with their effects on efflux and biofilm formation. Modulation of bacterial communication signals represents a complementary strategy to reduce the expression of resistance and virulence genes without directly inducing the selection of resistant strains [18].

Overall, terpenes and terpenoids act on multiple fronts to counteract antimicrobial resistance: they inhibit efflux pumps, impair biofilm formation, alter cell membrane integrity, and potentially interfere with quorum sensing. Their synergistic properties with conventional antibiotics make them promising candidates for combined therapeutic strategies aimed at overcoming bacterial resistance mechanisms.

### 2.4. Natural Antimicrobial Peptides (NAMPs)

Natural antimicrobial peptides (NAMPs), also known as host defense peptides, are an evolutionarily conserved class of small bioactive proteins present in all living organisms (plants, animals, fungi, and bacteria) that play a fundamental role in innate immunity and protection against infections. These peptides, typically amphipathic and positively charged, interact with microbial components such as membranes and biofilms in ways that make them promising agents against antimicrobial resistance.

A primary mechanism by which NAMPs exert their activity is the disruption of the microbial cell membrane, mediated by electrostatic interactions between the positively charged AMPs and the negatively charged bacterial membranes. This interaction leads to membrane destabilization, pore formation, and cell lysis. Such a mechanism reduces the likelihood of resistance development compared with traditional antibiotics, as it targets structurally conserved components of the microbial cell [19].

In addition to direct membrane disruption, NAMPs can inhibit biofilm formation and persistence. Recent systematic reviews highlight that many NAMP families are capable of degrading biofilms or preventing their maturation, thereby enhancing antibiotic penetration and efficacy even against multidrug-resistant strains [20].

Another relevant aspect of antimicrobial peptides is their ability to modulate quorum sensing and gene regulation associated with virulence and resistance. Certain NAMPs (such as melittin) not only directly target bacteria but can also interfere with chemical signals that regulate the expression of resistance and efflux genes within biofilms, contributing to the weakening of community cohesion and bacterial survival in the presence of antibiotics [21].

Finally, compared with conventional antibiotics, many NAMPs exhibit broad-spectrum activity against Gram-positive and Gram-negative bacteria, fungi, and other pathogens, often with a low propensity for resistance development and potential synergy with standard therapies. These characteristics make natural antimicrobial peptides ideal candidates for the development of novel small-molecule inhibitors and combined therapeutic strategies aimed at overcoming the challenges posed by antimicrobial resistance [22].

### 2.5. Microbial Secondary Metabolites

Microbial secondary metabolites represent one of the oldest and most productive sources of antimicrobial agents in modern clinical practice. Historically, many of the most important antibiotics—such as penicillin, streptomycin, vancomycin, and other natural derivatives—originate from soil microorganisms, including fungi of the genus Penicillium and bacteria of the genus Streptomyces, which produce bioactive compounds to compete with other microorganisms in their natural environment. These secondary metabolites are not essential for the growth of the producing organism but play fundamental ecological roles in defense and competition, forming the cornerstone of antibiotic discoveries throughout the twentieth century and beyond.

In particular, numerous compounds isolated from marine bacteria have demonstrated activity against multidrug-resistant pathogens, including methicillin-resistant Staphylococcus aureus (MRSA), methicillin-resistant *Staphylococcus epidermidis* (MRSE), vancomycin-resistant enterococci (VRE), multidrug-resistant *Mycobacterium tuberculosis* (MDR-TB), and amphotericin B-resistant *Candida albicans.* Moreover, these compounds may exert indirect antibacterial activity by influencing biofilm formation [23].

In addition, the production of secondary metabolites within microbial communities can modulate antibiotic tolerance and resistance through effects on bacterial physiology and the expression of resistance genes. Some of these metabolites may induce stress responses or alter microbial susceptibility to conventional antibiotics, highlighting a complex and bidirectional role in the dynamics of microbial resistance [24].

Overall, microbial secondary metabolites represent a strategic resource for the discovery of new antimicrobial agents and therapeutic adjuvants, with applications ranging from the direct inhibition of resistant pathogens to the modulation of resistance mechanisms such as biofilm formation and antibiotic tolerance.

Table 1 provides a comprehensive overview of the main classes of natural compounds discussed, including their representative examples, anti-AMR mechanisms, effects on resistance phenotypes, and potential therapeutic roles.

### 2.6. Synthetic and Semisynthetic Small-Molecule Inhibitors Against Antimicrobial Resistance

In addition to the natural compounds discussed in the previous sections, a distinct class of agents against antimicrobial resistance includes synthetic or semisynthetic small-molecule inhibitors, rationally designed to interfere with specific resistance mechanisms rather than acting as traditional bactericidal antibiotics. These inhibitors are being investigated as therapeutic adjuvants to restore or enhance the efficacy of existing antibiotics and to target critical molecular determinants associated with antimicrobial resistance.

One of the most advanced strategies in this field involves β-lactamase inhibitors, enzymes produced by many Gram-negative pathogens that inactivate β-lactam antibiotics. Molecules such as avibactam, vaborbactam, and relebactam have been developed to bind with high affinity to β-lactamases in resistant bacteria, thereby restoring the activity of antibiotics such as ceftazidime and meropenem. This approach demonstrates how small-molecule inhibitors can be integrated into pharmacological combinations to overcome complex resistance mechanisms in clinical pathogens [25,26,27].

Another important class of small-molecule inhibitors comprises bacterial efflux pump inhibitors (EPIs), designed to block active transport systems responsible for expelling antibiotics and other therapeutic molecules from the bacterial cell. Overexpression of efflux pumps represents one of the most widespread resistance mechanisms, as it reduces intracellular drug concentrations below therapeutic levels and contributes to the development of multidrug-resistant phenotypes, biofilm formation, and cellular persistence [28]. EPIs generally lack intrinsic antibacterial activity but function as adjuvants by restoring antibiotic susceptibility through increased intracellular drug accumulation. A widely studied example is phenylalanine-arginine β-naphthylamide (PAβN), a competitive inhibitor of Resistance-Nodulation-Division (RND) family pumps, capable of potentiating the activity of fluoroquinolones, β-lactams, and tetracyclines against *Pseudomonas aeruginosa* and other Gram-negative bacteria. Experimental evidence further indicates that efflux inhibition may reduce biofilm formation and the expression of virulence factors, suggesting a dual “resistance-breaking” and antivirulence effect [29,30].

In parallel, some small-molecule inhibitors are designed to block quorum sensing, a strategy that can reduce the expression of virulence factors and biofilm formation, both associated with resistant phenotypes [31]. Significant examples of small-molecule inhibitors with such potential include halogenated furanones synthesized based on molecules isolated from the marine alga Delisea pulchra, which exhibit inhibitory activity against Autoinducer-2, a signaling molecule involved in quorum sensing [32], as well as ligands targeting quorum-sensing receptors such as LasR and RhlR in *Pseudomonas aeruginosa* [33,34,35].

Finally, emerging approaches involve small-molecule inhibitors capable of modulating evolutionary processes associated with the emergence of resistance, such as the SOS response, DNA repair pathways, and antibiotic-induced stress mutagenesis. Inhibition of these pathways may reduce the frequency of adaptive mutations and slow the acquisition of resistance during treatment, representing a complementary preventive strategy alongside conventional antibiotics [36,37].

Overall, synthetic or semisynthetic small-molecule inhibitors represent an important class of functional molecules in the fight against antimicrobial resistance, acting on specific molecular targets and often integrating with conventional antibiotics to sustain therapeutic efficacy and limit the spread of resistant phenotypes.

Table 2 summarizes the main classes of synthetic and semisynthetic small-molecule inhibitors, highlighting their mechanisms, effects on resistance and therapeutic potential as antibiotic adjuvants.

## 3. Materials and Methods

### 3.1. Study Design

The present work is a narrative review aimed at identifying and analyzing the available evidence on the role of natural compounds and synthetic or semisynthetic small-molecule inhibitors as potential strategies against antimicrobial resistance. The review specifically focused on mechanisms involved in resistance modulation rather than solely on direct antimicrobial activity.

### 3.2. Literature Search Strategy

The literature search was conducted, in January 2026, using PubMed and included articles published up to 2025. Combinations of keywords and MeSH terms were employed. The main search terms included: “antimicrobial resistance,” “antibiotic resistance,” “natural compounds,” “phytochemicals,” “polyphenols,” “alkaloids,” “terpenes,” “terpenoids,” “natural antimicrobial peptides,” “microbial secondary metabolites,” “efflux pump inhibition,” “biofilm inhibition,” “quorum sensing,” “antibiotic adjuvants,” and “resistance-modifying agents.”

Search strings were combined using Boolean operators (AND/OR) to maximize both sensitivity and specificity of the results.

### 3.3. Inclusion and Exclusion Criteria

The literature search was restricted to articles published in peer-reviewed journals and written in English. Both original research articles and review papers were considered if they provided mechanistic insights into antimicrobial resistance modulation or antibiotic adjuvant activity. Studies focusing exclusively on antimicrobial activity without analysis of anti-resistance mechanisms were excluded. No strict restrictions were applied regarding study design or species in order to capture a broad range of experimental evidence related to antimicrobial resistance mechanisms.

### 3.4. Study Selection and Data Synthesis

Following an initial screening based on title and abstract, potentially relevant articles were assessed in full text. Extracted information included: class of natural compound, origin, anti-AMR mechanism, pathogens studied, type of experimental evidence, and potential application as an antibiotic adjuvant.

The results of the review were subsequently organized according to classes of natural compounds, and for each class the main mechanisms involved in counteracting antimicrobial resistance were synthesized (efflux pump inhibition, biofilm interference, quorum sensing modulation, and membrane permeability alteration).

### 3.5. Structure of the Review

In order to ensure a clear and systematic synthesis of the collected evidence, the compounds included in the review were organized primarily according to chemical-structural and biosynthetic criteria, grouping natural molecules based on their biological origin and shared molecular characteristics. Specifically, natural products were classified into the following categories: polyphenols, alkaloids, terpenes and terpenoids, natural antimicrobial peptides, and microbial secondary metabolites. This classification allowed comparison of compounds within the same chemical family in terms of pharmacological properties, mechanisms of action, and potential role in counteracting antimicrobial resistance.

After discussing natural compounds grouped according to their chemical structure and biological origin, a separate section was dedicated to synthetic or semisynthetic small-molecule inhibitors, defined as low-molecular-weight agents rationally designed to target specific antimicrobial resistance mechanisms.

This methodological distinction allowed a clear separation between compounds derived from natural sources and molecules designed or optimized through medicinal chemistry approaches. The latter category included efflux pump inhibitors, β-lactamase inhibitors, quorum sensing modulators, and other adjuvants capable of restoring antibiotic susceptibility.

This thematic organization, based on a combination of structural and functional criteria, was adopted to reduce conceptual overlap among classes, facilitate comparative interpretation of anti-resistance mechanisms, and provide a coherent framework of the various natural and synthetic strategies currently being investigated as complementary approaches to conventional antibiotics.

## 4. Conclusions

The increasing spread of antimicrobial resistance represents one of the major threats to global public health, making the identification of complementary or alternative therapeutic strategies to conventional antibiotics an urgent priority. In this context, the present review systematically analyzed two distinct yet complementary pharmacological approaches: on the one hand, natural compounds, classified according to their chemical structure and biological origin; on the other, synthetic or semisynthetic small-molecule inhibitors, rationally designed to target specific resistance mechanisms.

Natural compounds—including polyphenols, alkaloids, terpenes and terpenoids, antimicrobial peptides, and microbial secondary metabolites—exhibit a broad spectrum of anti-AMR activities, often characterized by multitarget mechanisms such as membrane disruption, inhibition of essential enzymes, modulation of efflux pumps, interference with biofilm formation, and attenuation of virulence. Their high structural and biological diversity makes them a valuable source of lead compounds and adjuvant molecules, with the potential advantage of exerting lower selective pressure compared with traditional bactericidal antibiotics. However, limitations such as poor physicochemical stability, low bioavailability, variability in extraction processes, and challenges in standardization may hinder their clinical translation.

In parallel, synthetic and semisynthetic small-molecule inhibitors represent a more targeted and rational approach, based on the identification of specific molecular determinants involved in resistance mechanisms. β-lactamase inhibitors, efflux pump modulators, quorum-sensing antagonists, and molecules capable of interfering with evolutionary processes underlying resistance demonstrate that it is possible to restore the efficacy of existing antibiotics or reduce bacterial pathogenicity without necessarily exerting direct bactericidal effects. Compared with natural products, these molecules generally offer greater structural controllability, pharmacokinetic optimization, and manufacturing reproducibility, thereby facilitating preclinical and clinical development.

From a translational perspective, several resistance-modifying strategies discussed in this review have already reached advanced stages of clinical development. β-lactamase inhibitors such as avibactam, vaborbactam, and relebactam are currently used in combination therapies with β-lactam antibiotics and represent successful examples of small-molecule inhibitors designed to restore antibiotic efficacy in clinical settings. In parallel, several antimicrobial peptides and their synthetic derivatives are undergoing preclinical evaluation or early-phase clinical trials as potential alternatives or adjuncts to conventional antibiotics. For instance, pexiganan, a magainin analog, has been investigated in clinical trials for the treatment of infected diabetic foot ulcers; omiganan, an indolicidin analog, has been evaluated in clinical studies for severe papulopustular rosacea; C16G2, a novispirin analog, has been explored in early clinical trials for the prevention of dental caries caused by *Streptococcus mutans* [38]. These examples highlight the growing interest in antimicrobial peptides as translational candidates capable of complementing conventional antibiotic therapy and addressing antimicrobial resistance through alternative mechanisms of action.

Taken together, current evidence suggests that the integration of natural compounds and synthetic adjuvant molecules may represent the most promising strategy to counteract AMR. Antibiotic–adjuvant combinations, antivirulence therapies, and “resistance-breaking” strategies could prolong the clinical lifespan of currently available antibiotics, reduce required dosages, and limit the selection of multidrug-resistant strains.

Future research directions should focus on the identification of novel molecular targets, the application of rational drug design and high-throughput screening technologies, and the evaluation of efficacy and safety in in vivo and clinical models. Moreover, from a public health perspective, these pharmacological strategies must be integrated with antibiotic stewardship programs, epidemiological surveillance, and infection prevention measures.

In conclusion, both natural products and rationally designed small-molecule inhibitors represent complementary resources in the fight against antimicrobial resistance. Their synergistic development could significantly contribute to the establishment of new sustainable therapeutic options and to the preservation of antibiotic efficacy for future generations.

## Figures and Tables

**Table 1:** Overview of natural compound classes.

Compound Class	Examples	Anti-AMR Mechanisms	Effects on Resistance Phenotypes	Potential Therapeutic Roles	References
Polyphenols	Quercetin, Curcumin, Luteolin, EGCG, Proanthocyanidins, Ellagic acid	Efflux pump inhibition; biofilm disruption; quorum sensing modulation; membrane permeability alteration	Increased intracellular antibiotic concentration; reduced virulence; improved antibiotic penetration	Antibiotic adjuvants; antivirulence agents	[9,10,11]
Alkaloids	Reserpine, Piperine, Berberine	Efflux pump inhibition; replication impairment; biofilm interference; quorum sensing modulation	Reduced MIC of antibiotics; impaired bacterial replication; decreased biofilm persistence	Combination therapy enhancers	[12,13]
Terpenes & Terpenoids	Carvacrol, Thymol, Limonene, Carvone, β-citronellol	Efflux pump inhibition; biofilm inhibition; membrane destabilization; quorum sensing interference	Increased membrane permeability; reduced biofilm biomass; enhanced antibiotic susceptibility	Synergistic co-therapies	[14,15,16,17,18]
Natural antimicrobial peptides (NAMPs)	Melittin	Membrane disruption; biofilm interference; quorum sensing modulation	Rapid bacterial killing; reduced resistance emergence; improved antibiotic penetration	Direct antimicrobial agents; adjuvant therapy	[19,20,21,22]
Microbial secondary metabolites	Marine-derived compounds	Direct antimicrobial activity; biofilm modulation; stress response modulation	Activity against MDR pathogens; interference with tolerance mechanisms	Novel antibiotic scaffolds; resistance modulators	[23,24]

**Table 2:** Overview of synthetic and semisynthetic small-molecule inhibitors.

Compound Class	Examples	Anti-AMR Mechanisms	Effects on Resistance Phenotypes	Potential Terapeutic Roles	References
β-lactamase inhibitors	Avibactam, Vaborbactam, Relebactam	Inhibition of β-lactamases	Restoration of β-lactam efficacy	Fixed antibiotic combinations	[25,26,27]
Efflux pump inhibitors	PAβN, other RND inhibitors	Inhibition of efflux pumps	Increased intracellular drug accumulation	Antibiotic adjuvants	[29,30]
Quorum sensing inhibitors	Halogenated furanones; LasR/RhlR antagonists	Blockade of signaling pathways regulating virulence and biofilm	Reduced virulence expression; impaired biofilm formation	Antivirulence strategy	[31,32,33,34,35]
Anti-evolution drugs	SOS response inhibitors; DNA repair inhibitors	Inhibition of stress-induced mutagenesis and adaptive evolution	Reduced emergence of resistance during therapy	Resistance-prevention strategy	[36,37]

## Data Availability

No new data were created or analyzed in this study. Data sharing is not applicable to this article.
